# Assessment of Construct Validity and Reliability of the Hospital Anxiety and Depression Scale in Greek Couples With Infertility Undergoing In Vitro Fertilization Cycles

**DOI:** 10.7759/cureus.72350

**Published:** 2024-10-25

**Authors:** Meropi Moutzouri, George Koulierakis, Antigoni Sarantaki, Kleanthi Gourounti

**Affiliations:** 1 Department of Midwifery, Faculty of Health and Care Sciences, University of West Attica, Athens, GRC; 2 Department of Public Health Policy, Laboratory of Epidemiology, Health Determinants and Well-Being, Division of Epidemiology, Prevention and Quality of Life, University of West Attica, Athens, GRC

**Keywords:** anxiety, confirmatory factor analysis, depression, infertile couple, infertility, in vitro fertilization (ivf), reliability, validity

## Abstract

Introduction: The Hospital Anxiety and Depression Scale (HADS) is a widely used screening instrument created to assess anxiety and depression symptoms during the experience of various health problems. It has also been increasingly applied to populations facing infertility. The current cross-sectional study aimed to assess the construct validity and reliability of the HADS in a sample of Greek participants undergoing in vitro fertilization (IVF) cycles.

Materials and methods: This study included 90 couples with infertility referred to an assisted reproduction unit in Heraklion, Greece, and an infertility center in Athens, Greece. To validate the scale, confirmatory factor analyses (CFA) were performed. Several goodness-of-fit indices were utilized including the comparative fit index (CFI), the root mean squared error of approximation (RMSEA), the standardized root mean square residual (SRMR), and normed chi-square index with the degree of freedom (c^2^/df). The reliability analysis was conducted by calculating Cronbach's alpha.

Results: The confirmatory factor analysis revealed that all of the four fitness indexes were good (x^2^/df=1.489, CFI=0.943, RMSEA=0.052, SRMR=0.055). Based on the item-scale correlation coefficients, reliability was very good for the anxiety factor (alpha = 0.808), good for the depression factor (alpha = 0.707), and very good for the total questionnaire (alpha = 0.858).

Conclusion: The results of confirmatory factor analyses and reliability analyses proved that HADS met the criteria of construct validity and reliability, making it suitable for use with couples undergoing an IVF cycle. Examining negative emotions, such as anxiety and depression, during the period of fertility problems and its treatment is important to evaluate the psychological maladjustment of couples with infertility and to create psychological interventions by health professionals that help individuals with infertility manage distress during IVF.

## Introduction

Couples with infertility show a satisfactory psychological adjustment to assisted reproduction methods and to an unsuccessful cycle of in vitro fertilization (IVF), yet a group of individuals with infertility face emotional difficulties and psychopathology [1,2]. Thus, it is crucial to identify subgroups of couples with infertility who suffer from emotional distress, such as anxiety and depression, especially during the initial stages of an IVF cycle, and provide psychological support and counseling.

Α benefit of the Hospital Anxiety and Depression Scale (HADS), a common self-report screening tool, measuring emotional symptoms during the experience of a health threat [3,4] is the conciseness, making it suitable for identifying and quantifying anxiety and depression symptoms of general hospital patients who require additional psychiatric evaluation and support [5,6]. It is not used to identify the presence of anxiety and depression or to diagnose psychiatric disorders. The tool has been translated and extensively utilized in over 25 countries since it was first developed [6]. Its well-proved reliability and validity have been demonstrated by extended reviews of Wu et al. [6] and Nikolovski et al. [7].

Several studies indicate a two-factor structure of the HADS. The study of Mykletun et al., which included 51,930 participants and is the largest in the literature, confirmed that a bidimensional structure for the questionnaire is correct [8]. Additionally, Lloyd et al. supported the original two-factor structure [9]. The Greek version of the HADS also appeared to have a two-dimensional structure, suggesting that the two subscales of the HADS independently assess anxiety and depression [10].

The HADS has been utilized to assess anxiety and depression in patients suffering from a variety of health conditions such as cancer [11], human immunodeficiency virus (HIV) [12], and rheumatoid arthritis [13]. The scale has also been used in individuals with infertility [14-16]. The HADS has been translated into Greek and successfully validated in a palliative care unit for cancer patients [17] and in general hospital patients [10].

To the best of our knowledge, the psychometric properties of the Greek version of the HADS have never been evaluated in couples with infertility. Thus, the aim of the current study was to assess the construct validity and internal consistency reliability of the tool in a sample of Greek couples undergoing an IVF cycle.

## Materials and methods

Study design and setting

This was a cross-sectional study that assessed the psychometric properties of the HADS Greek version in a sample of couples diagnosed with infertility. The participants were recruited from the assisted reproduction unit of the University General Hospital of Heraklion (PAGNI), Heraklion, Greece (February to April 2014) and the infertility center of the Maternity Hospital Elena Venizelou, Athens, Greece (June 2023). These hospitals provide various infertility and fertilization treatments.

Sample calculation and selection

The sample of the current study was calculated based on the overall number of variables used [18,19]. Previous analyses revealed that the largest linear regression included seven independent variables, thus, a sample size of 80 couples was considered sufficient. An extra 30% (24 couples) was added to the required sample size to substitute potential dropouts and incomplete questionnaires.

In order to participate, individuals had to: (a) have the ability to speak and read Greek, (b) have been undergoing an IVF cycle, and (c) be at least 18 years old. A random sampling method was utilized. More specifically, the secretary of each infertility unit gave the principal investigator (MM) a list of couples with infertility who were undergoing an IVF cycle. The investigator then approached all the couples on these lists, briefly explaining the study's objectives and procedures, and invited them to participate. Couples who agreed to participate were then assessed for eligibility according to the previously mentioned criteria.

Of the final sample of 208 eligible individuals, 28 participants withdrew from the study, thus leaving a total of 90 couples (180 individuals), who completely filled out the questionnaire.

Data collection

Demographic/clinical characteristics including gender, age, and previous IVF cycles were collected. Data on anxiety and depression was collected with the use of HADS.

The HADS is a brief self-reporting tool designed for the assessment of anxiety and depression symptoms in non-psychiatric populations in medical settings. The scale consists of 14 questions. Of these, seven measure anxiety and constitute the anxiety subscale (HADS-A) (e.g. I can sit at ease and feel relaxed), and the remaining seven measure depression and constitute the depression subscale (HADS-D) (e.g. I still enjoy the things I used to enjoy). Each question receives one answer out of four possible responses in a Likert scale format with a scoring range of 0-3, resulting in a possible score ranging from 0 to 21 for anxiety and 0 to 21 for depression. Higher scores indicate greater anxiety and depression.

In this study, the Greek version of HADS translated by Mystakidou et al. [17] was used after permission was granted. In order to minimize the examiner’s involvement during the participants’ completion of the HADS and ensure homogeneity of the results, the scale was administered and scored by only the first author (MM).

Ethical consideration

The study was approved by the Ethics Committee of PAGNI and the Ethics Committee of Elena Venizelou Hospital (approval numbers: 4342/10-4-2013 and 19229/22-9-2022, respectively). Eligible participants who agreed to join the study were fully informed about its purpose, the potential risks and benefits of participation, and assured of the confidentiality of their data through a consent form, which was obtained from each participant before data collection began.

Statistical analysis

To confirm the validity of the two-factor (anxiety and depression) construct of the HADS, confirmatory factor analysis (CFA) was conducted using the IBM SPSS Amos™, Version 22.0 (Released 2018; IBM Corp., Armonk, New York, United States). The primary goal of the CFA was to ensure the models' good fit with minimal modifications to the structure and composition of the factors. The criteria for retaining individual questions in the model were the statistical significance of their linear relationship with the corresponding latent factor and a minimum standardized regression weight (Std B) to be set at 0.4 [20]. If a question did not meet either or both of these criteria, it was removed. In cases where the significance criterion was met but the Std B coefficient was below 0.4, its removal was decided after considering additional criteria, such as the overall model fit and the reliability of the factor to which the question belonged.

A series of fit indices were examined to evaluate the quality of model fit, including comparative fit index for sample size (CFI greater than 0.90) [21], normed chi-square index with the degree of freedom (a less than three c2/df indicated a good fit) [22], residual mean square error which is the square root of the mean of the estimation error and describes the expected adequacy of the model if it were estimated in the actual population rather than a sample (root mean squared error of approximation (RMSEA) less than 0.06) [21] and standardized root mean squared residual (SRMR) less than 0.08 [23,24].

After completing the CFA and ensuring structural validity, the HADS and its subscales were tested for internal consistency reliability by evaluating the Cronbach's alpha value, with a minimum acceptable alpha value set at 0.7. The reliability analysis was conducted using IBM SPSS Statistics for Windows, Version 26.0 (Released 2019; IBM Corp.).

## Results

Participant characteristics

The total number of respondents undergoing IVF cycles who met the eligible criteria and participated in the study was 180 (90 men and 90 women). The mean age of the respondents was 40.59 years (SD = 9.07) and the average number of previous IVF cycles was 1.55 (SD = 2.89). The mean HADS-A and HADS-D subscale scores for participants were 6.64 (SD = 3.79) and 3.44 (SD = 2.85), respectively (Table 1).

**Table 1:** The mean demographic/clinical characteristics of the participants and the mean HADS-A and HADS-D scores HADS: Hospital Anxiety and Depression Scale; HADS-A: HADS anxiety subscale; HADS-D: HADS depression subscale; IVF: in vitro fertilization

Variables	Values, mean ±SD
Age (years)	40.59±9.07
Number of previous IVF cycles	1.55 ±2.89
HADS-A score	6.64±3.79
HADS-D score	3.44±2.85

Internal consistency

The Cronbach’s alpha value for the total HADS was 0.858 suggesting very good consistency and showing that the 14 items of the questionnaire were read and answered by the participants equally reliably (Table 2). Additionally, the seven items of the anxiety subscale (HADS-A) were read and answered by the participants equally reliably, with a Cronbach’s alpha of 0.808. The depression subscale (HADS-D) was measured reliably too, with a Cronbach’s alpha of 0.707 (Table 2). As a result, Cronbach’s alpha values for HADS-A and HADS-D indicated very good and good internal consistency, respectively.

**Table 2:** Reliability analysis of the HADS and its subscales HADS-A and HADS-D HADS: Hospital Anxiety and Depression Scale; HADS-A: HADS anxiety subscale; HADS-D: HADS depression subscale

HADS items	Cronbach's α
HADS	0.858
HADS-A	0.808
HADS-D	0.707

CFA

**Figure 1:**
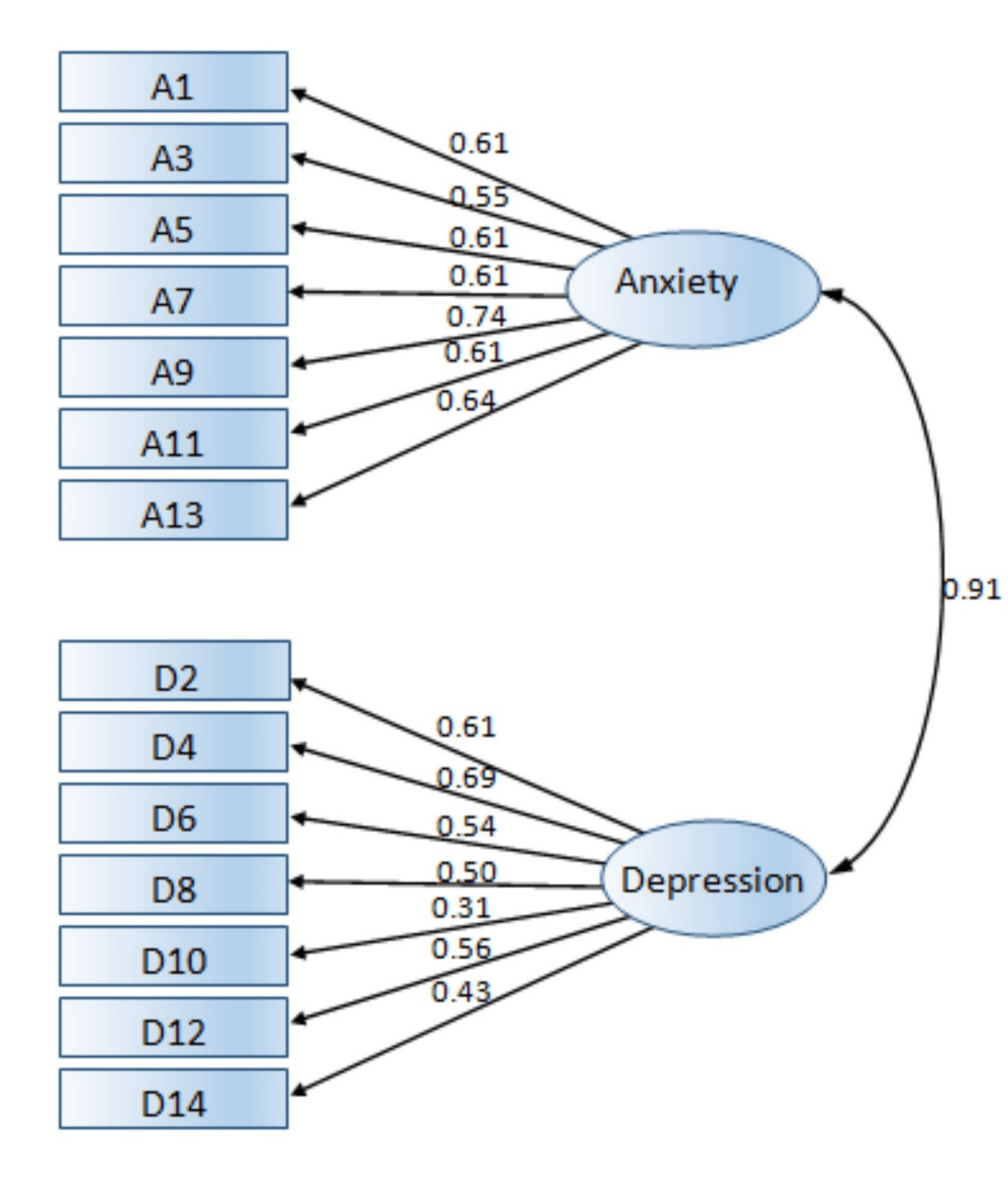
The confirmatory factor analysis for the Greek version of HADS HADS: Hospital Anxiety and Depression Scale

HADS has a two-factor structure; factor HADS-A for anxiety and factor HADS-D for depression (Table 3). The CFA was applied to the two subscale items of the HADS (Figure 1). All the factor loadings ranged from 0.31 to 0.74. The standardized regression coefficients between the two subscales and their parent latent factors are presented in Table 4. As can be seen, all linear regressions between the questions and the latent factors were statistically significant (p<0.05), and all Std B coefficients were above the threshold of 0.4, except for the coefficient of question D10, which was at 0.311. Given that this coefficient was significantly lower than the 0.4 threshold, the overall fit of the model was examined first to decide whether to retain the question in the model.

**Table 3:** The HADS item numbers and texts HADS: Hospital Anxiety and Depression Scale; HADS-A: HADS anxiety subscale; HADS-D: HADS depression subscale * Items that were reverse scored before summation.

Item number	Item text
HADS-A	
A1	I feel tense or wound up. *
A3	I get a sort of frightened feeling as if something awful is about to happen. *
A5	Worrying thoughts go through my mind. *
A7	I can sit at ease and feel relaxed.
A9	I get a sort of frightened feeling like 'butterflies' in the stomach.
A11	I feel restless as if I have to be on the move. *
A13	I get sudden feelings of panic. *
HADS-D	
D2	I still enjoy the things I used to enjoy.
D4	I can laugh and see the funny side of things.
D6	I feel cheerful. *
D8	I feel as if I am slowed down. *
D10	I have lost interest in my appearance. *
D12	I look forward with enjoyment to things.
D14	I can enjoy a good book or TV program.

**Table 4:** Linear regressions of the confirmatory factor analysis structural model of the HADS HADS: Hospital Anxiety and Depression Scale; B: beta coefficient; Std B: standardized beta; S.E.: standard error; C.R.: critical ratio

	B	Std B	S.E.	C.R.	P-value
A1	1.137	0.609	0.165	6.870	< 0.001
A3	1.049	0.552	0.166	6.329	< 0.001
A5	1.245	0.609	0.181	6.874	< 0.001
A7	1.005	0.607	0.147	6.857	< 0.001
A9	1.074	0.735	0.135	7.957	< 0.001
A11	1.087	0.610	0.158	6.878	< 0.001
A13	1.000	0.637			
D2	1.239	0.608	0.255	4.854	< 0.001
D4	1.855	0.690	0.364	5.097	< 0.001
D6	1.157	0.539	0.252	4.597	< 0.001
D8	1.152	0.497	0.261	4.413	< 0.001
D10	0.959	0.311	0.293	3.272	0.001
D12	1.302	0.560	0.278	4.681	< 0.001
D14	1.000	0.427			

Based on the goodness-of-ft indices of the model (Table 5), the x²/df index was 1.489, which was significantly lower than the strict threshold of 5, the CFI was 0.943 which showed a very good fit and above the threshold of 0.9, the RMSEA was 0.052 which indicated an excellent fit, and the SRMR was 0.055 which was below the desired upper limit of 0.08. As a result, all indices indicated a good fit between the model and the data.

**Table 5:** The goodness-of-ft indices of the model x^2^: Chi-square index; df: degree of freedom; x^2^/df: normed Chi-square index with the degree of freedom; CFI: comparative fit index; RMSEA: root mean squared error of approximation; SRMR: standardized root mean square residual

The goodness-of-fit indices	x^2^	df	x^2^/df	CFI	RMSEA	SRMR
Value	113.165	76	1.489	0.943	0.052	0.055

## Discussion

This was the first study to assess the internal consistency and the validity of the Greek version of the HADS in couples with infertility. According to the results, the HADS met the criteria for internal consistency and construct validity. Both separate subscales of the HADS and the total questionnaire demonstrated good to very good Cronbach’s alpha values for internal consistency. These psychometric properties of the Greek version of the HADS on couples with infertility are similar to those of Greek general hospital patients in the study of Michopoulos et al. [10] and those of Greek cancer patients in the research of Mystakidou et al. [17]. Furthermore, these satisfactory values are consistent with those reported by Amini et al. [25], Biringer et al. [26], Matsubayashi et al. [27] and Anderson et al. [28] during the experience of infertility and its treatment.

Although several studies have utilized the HADS to report the percentage of anxious and depressed infertile populations, they did not evaluate the structure of the HADS [29,30]. In the present study, factor analysis indicated acceptable goodness-of-ft indices of the two-structure model in the case of individuals undergoing IVF cycles. This result aligns with what was reported by Matsubayashi et al. [29]. 

Based on the good psychometric properties of the scale, the HADS can be used for screening psychological symptoms during the experience of infertility and an IVF cycle. The consistent use of HADS in future research on couples with infertility could facilitate global comparisons of the prevalence of psychological disorders in this population. Moreover, examining anxiety and depression during infertility and IVF is crucial for assessing the psychological maladjustment of such couples. This evaluation can help health professionals, such as psychologists, to develop interventions in the counseling portion of an IVF cycle that assist individuals with infertility in managing distress during IVF.

Strengths and limitations

The current study was the first and the only one that examined the reliability and construct validity of a widely used scale in a specific population in Greece. Despite its novelty, the current study had several limitations that should be considered. Firstly, diagnostic interviews were not conducted, preventing any analysis of the scale's sensitivity and specificity. Moreover, the test-retest reliability of the scale was not assessed. Additionally, although we evaluated individuals with infertility undergoing IVF, we did not gather information on their emotional status before starting assisted reproduction; thus, future studies need to examine the long-term evolution of anxiety or depressive symptoms in this population. Finally, the sample was collected in two different time phases, which were a few years apart from each other.

## Conclusions

The findings of the present study suggest that the Greek version of HADS is reliable and valid for use in Greek couples during the experience of infertility and IVF cycles. It could be used in infertility centers in order to identify anxiety and depression in individuals who need psychological intervention or psychiatric care. The doctors, midwives, and IVF nurse practitioners, who are counseling the couple regarding their fertility problem, must be familiar with the HADS to assist in the identification of couples at high risk of psychological distress.
